# Microglial activation and hypothalamic structural plasticity in HFD obesity: insights from semaglutide and minocycline

**DOI:** 10.1016/j.jlr.2024.100736

**Published:** 2024-12-24

**Authors:** Xi Rong, Fang Wei, Yuqi Jiang, Qintao Ma, Dongmei Wang, Jie Shen

**Affiliations:** 1 Department of Endocrinology and Metabolism, Shunde Hospital of Southern Medical University (The First People’s Hospital of Shunde Foshan), Foshan, Guangdong Province, China; 2 Department of Geriatric Endocrinology, The First Affiliated Hospital of Guangxi Medical University, Nanning, Guangxi Zhuang Autonomous Region, China

**Keywords:** microglial activation, hypothalamic structural plasticity, high-fat diet obesity, semaglutide, minocycline

## Abstract

High-fat diet (HFD)-induced microglial activation contributes to hypothalamic inflammation and obesity, but the mechanisms linking microglia to structural changes remain unclear. This study explored the role of microglia in impairing hypothalamic synaptic plasticity in diet-induced obesity mice and evaluated the therapeutic potential of semaglutide (Sema) and minocycline (MI). Six-week-old C57BL/6J mice were divided into low-fat diet and HFD groups. At week 30, the HFD-fed mice were treated daily with Sema or MI for six weeks. Confocal microscopy assessed hypothalamic dendritic spines, synaptic organization, and microglia–synapse interactions. We also analyzed microglial morphology, CD68/CD11b colocalization with Iba-1, synaptic marker expression, and phagocytosis-related pathways (C1q, C3, CD11b). BV2 microglia were used to examine the direct effects of Sema or MI on microglia and validate the in vivo findings. HFD feeding induced microglial activation, as indicated by increased colocalization of CD68 or synaptophysin and CD11b with Iba-1, along with elevated C1q, C3, and CD11b expression, signaling enhanced synaptic phagocytosis. This was accompanied by reduced hypothalamic dendritic spines, decreased synaptic marker expression, and disrupted excitatory/inhibitory synaptic organization in the melanocortin system, as well as impaired glucose metabolism, disrupted leptin-ghrelin balance, and increased food intake and body weight. Sema and MI treatments reversed the pathological changes of microglial activation and restored hypothalamic synaptic structure, although their effects on synaptic organization and metabolic outcomes differed. Our findings highlight the key role of microglial activation in hypothalamic synaptic impairment in diet-induced obesity models, with Sema and MI possibly offering distinct therapeutic pathways to mitigate these impairments.

In modern society, the prevalence of a high-fat diet (HFD) has increased significantly due to changes in dietary habits and lifestyles. These diets, characterized by high caloric content, are major contributors to obesity. In addition to contributing to excessive caloric intake, a HFD negatively affects the central nervous system's ability to regulate feeding behaviors, specifically by disrupting hypothalamic feeding centers. This disruption exacerbates obesity progression and creates a vicious cycle of metabolic dysregulation. Therefore, understanding how HFD alters hypothalamic structure and function is critical for improving obesity interventions.

The hypothalamus, a critical brain region for regulating body weight and energy homeostasis, depends on the melanocortin system, particularly neuropeptide Y (NPY)/agouti-related peptide (AgRP) and proopiomelanocortin (POMC) neurons in the arcuate nucleus (ARC), to balance feeding behaviors and energy expenditure (1, 2, 3, 4). Within the hypothalamic melanocortin system, NPY/AgRP and POMC neurons not only sense nutrients and hormone signals but also receive input from other neurons through synaptic connections, including both excitatory and inhibitory inputs, which collectively shape feeding behavior regulation. Synaptic plasticity within the hypothalamus enables neurons to adapt their synaptic connections in response to nutrient and hormone signals, mediating dynamic changes in both neurotransmitter release and synaptic architecture (5, 6, 7). However, several studies have indicated that HFD consumption compromises hypothalamic synaptic plasticity, resulting in abnormal synaptic restructuring, disrupted excitatory-inhibitory balance, and reduced synaptic density in feeding-related centers (8, 9, 10, 11). HFDs have been shown to diminish synaptic inputs to NPY/AgRP and POMC neurons and increase glial coverage of their perikarya, highlighting the pivotal role of glial cells in synaptic reconfiguration (12, 13).

Microglia, the resident immune cells of the central nervous system (CNS), actively participate in immune responses and synaptic remodeling. They play crucial roles in antigen presentation, inflammation, and synaptic refinement through phagocytosis, affecting neuronal circuitry (14). Elevated free fatty acids from a HFD activate these immune cells in the brain, driving hypothalamic inflammatory responses and significantly contributing to obesity pathogenesis (15, 16, 17). Hyperactive microglia in the hippocampus can excessively engulf synapses, impair synaptic plasticity, and induce cognitive deficits in individuals with diet-induced obesity (DIO) (18, 19). This process involves complement components such as C1q and C3, which are among the classical "eat me" signals that tag synapses for removal, and microglial receptors such as CD11b, which facilitate synaptic engulfment (20, 21, 22). Despite studies demonstrating microglial roles in synaptic remodeling in other brain regions, their impact on hypothalamic synaptic plasticity during HFD-induced obesity, particularly concerning their phagocytic activity, remains unclear.

Some studies indicate that targeting microglial activity can influence obesity outcomes. Reducing microglial inflammatory responses to a HFD has beneficial effects in protecting against DIO, and attenuation of microglial phagocytic activity has been shown to reduce susceptibility to DIO (23, 24, 25, 26). Given the pivotal role of microglia in obesity, pharmacological interventions targeting these cells have garnered attention in the obesity field. Semaglutide (Sema), a GLP-1 receptor agonist (GLP-1RA), and minocycline (MI), a known microglial inhibitor, are known to modulate microglial activity and CNS inflammation. Studies have revealed that GLP-1RAs can modulate microglial activity and mitigate CNS inflammation, contributing to weight regulation (27, 28, 29). Similarly, MI has shown promise in attenuating weight gain in DIO models by inhibiting microglial activation (30).

Based on the well-established critical role of microglia in the pathogenesis of obesity, we hypothesized that a HFD triggers microglial activation and excessive synaptic pruning, impairing synaptic structural plasticity in the hypothalamus. Such pathological changes, including synaptic loss and disruption of the excitatory and inhibitory balance of melanocortin neurons, may ultimately contribute to the development of obesity. Pharmacological intervention with two compounds, Sema and MI, which both inhibit microglial activation and have weight-lowering effects, could restore hypothalamic synaptic plasticity impaired by HFDs.

To test this hypothesis, we induced DIO in mice with a HFD and activated BV2 microglia with palmitic acid (PA) in vitro. Sema (GLP-1RA) and MI (microglial inhibitor) were used to evaluate their therapeutic effects. We assessed synaptic plasticity and microglial activity, including dendritic spine density, synaptic organization, and phagocytosis-related proteins, to confirm microglial involvement in hypothalamic dysfunction and evaluate therapeutic efficacy.

## Materials and Methods

### Animals and diets

Weaned male C57BL/6J mice from SPF Biotechnology Company Limited (Beijing, China) were used. All procedures were ethically approved (approval No. 2023-S809-01) and followed animal care standards. The mice were fed either a HFD (D12492, Research Diets; 60% energy from fat, 5.24 kcal/g) or a low-fat diet (LFD) (D12450K, Research Diets; 10% energy from fat, 3.85 kcal/g). After one week of adaptation, 6-week-old mice were randomly assigned to four groups (n = 10): the LFD, HFD, Sema-treated, and MI-treated groups. LFD group mice received an LFD throughout the experiment, whereas the other groups were fed a HFD beginning at 6 weeks of age. Only male mice were used to avoid variability associated with the estrous cycle.

At age 30 weeks, the mice in the Sema and MI groups began receiving the corresponding pharmacological intervention in addition to being fed a HFD. The Sema group received daily intraperitoneal injections of Sema (31) (30 nM/kg/d, HY-114118, MedChemExpress), and the MI group received daily intraperitoneal injections of MI (30) (40 mg/kg/d, M9511-1G, Sigma‒Aldrich). Both the LFD and HFD groups received daily intraperitoneal injections of saline. The LFD group continued to receive a LFD, whereas the HFD group continued to receive a HFD. These interventions continued for an additional 6 weeks, with the entire experiment lasting 30 weeks for all the groups.

The mice were housed in plastic cages under controlled lighting conditions (12 h light/12 h dark). Food and water were available ad libitum. Fresh chow was provided daily, and daily food intake was measured by weighing the amount of food supplied and the amount of food left in the cage. Energy intake was estimated as the product of food consumption and the energy content of the diet.

### Intraperitoneal glucose tolerance test

The intraperitoneal glucose tolerance test (IPGTT) was performed on animals that were fasted for 6 h before and after pharmacological intervention. The animals received an intraperitoneal injection of glucose at a dosage of 1 g/kg body weight (20% glucose solution in sterile water). Blood samples were collected from the tail vein at 0, 15, 30, 60, and 120 min using a glucometer (Accu-Chek, Roche, Switzerland).

### Serum leptin and ghrelin assays

On the final day of the study, after 6 h of fasting, the animals were deeply anesthetized via an intraperitoneal injection of sodium pentobarbital (150 mg/kg). Serum leptin and total ghrelin levels were determined using commercial ELISA kits (leptin: ab100718, Abcam; ghrelin: BES1483K96T, BIOESN, China). The assays were conducted following the manufacturers’ instructions.

### Experimental setup for BV2 microglial culture and treatments

In this study, BV2 cells, a murine microglial cell line, were utilized. The cells were obtained from iCell Bioscience, Inc. (Shanghai, China). The culture medium was DMEM (KeyGEN BioTECH, China) supplemented with 10% fetal bovine serum, 1% penicillin‒streptomycin, and 2 mmol/L L-glutamine (Solarbio, Beijing, China). The cells were maintained at 37°C in a humidified incubator with 5% CO_2_.

Two experimental setups were established. In the first, BV2 cells were divided into four groups: *1*) control (untreated), *2*) PA (P5585, Sigma-Aldrich, induced microglial activation), *3*) MI (M9511-1G, Sigma‒Aldrich), and *4*) PA+MI (both PA and MI). In the second, the groups were *1*) control, *2*) PA, *3*) GLP-1RA (Sema, HY-114118, MedChemExpress), and *4*) PA+GLP-1RA (both PA and Sema).

### Cell survival assays

**supplemental Fig. S1:**
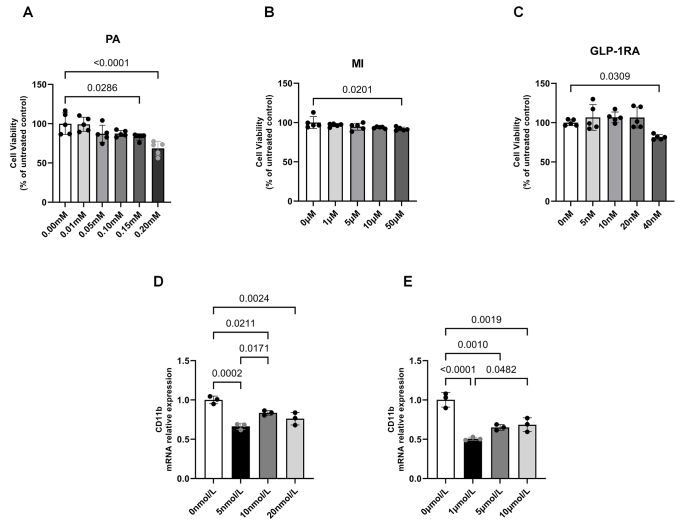
A–C: CCK assays were performed to assess the viability of BV2 microglia treated with various concentrations of palmitic acid (PA). The concentration of 0.1 mM PA was selected as the optimal dose to activate microglia, as determined by these results. Data were collected from 5 technical replicates. Statistical significance was determined using one-way ANOVA, followed by Dunnett’s multiple comparisons test. D–E: BV2 cells were pretreated with 0.1 mM PA to induce microglial activation, followed by intervention with different concentrations of semaglutide (D) or minocycline (E). The mRNA expression of *CD11b* was measured to evaluate the effectiveness of minocycline (MI) and semaglutide (GLP-1RA) in inhibiting PA-induced microglial activation. Statistical significance was determined using one-way ANOVA, followed by Tukey’s post hoc test (n = 3 independent experiments). CCK-8, cell counting kit-8; GLP-1RA, GLP-1 receptor agonist.

Cell viability was assessed using cell counting kit-8 (CCK-8) assay (KeyGEN BioTECH, China). BV2 microglia were seeded into 96-well plates at a density of 2 × 10^4^ cells per well and treated with various interventions. For the assay, 100 μl of fresh DMEM was added to each well, followed by the addition of 10 μl of CCK-8 solution. The plates were incubated at 37°C for 2 h, and cell viability was quantified by measuring the absorbance at 450 nm using a microplate reader (Thermo Fisher Scientific). To determine the optimal drug concentrations, preliminary experiments were conducted with a gradient of PA, MI, and Sema. On the basis of the cell viability and activation response, the final intervention concentrations were established as 0.1 mmol/L for PA, 1 μmol/L for MI, and 5 nmol/L for Sema. Details of these screening experiments are provided in the supplementary materials (supplemental Fig. S1).

### Phagocytic function assessment of BV2 microglial cells

Phagocytic activity of BV2 microglial cells was assessed using Protonex™ Green 500 Dextran (AAT Bioquest), a pH-sensitive fluorescent probe that fluoresces in acidic compartments like phagosomes and lysosomes, providing an indicator of phagocytosis. BV2 cells were cultured in 6-well plates and incubated with Protonex™ Green solution (100 μg/ml) for 30 min at 37°C. After incubation, cells were washed with PBS to remove excess dye. Fluorescence images were captured using a fluorescence microscope (CKX53, Olympus) to visualize acidic compartments. Phagocytic activity was quantified by calculating the mean fluorescence intensity, representing the degree of Protonex™ Green uptake. For quantitative analysis, the mean gray value (Mean) was determined by dividing the integrated optical density by the area (Area) of the region of interest. Data were expressed as the average fluorescence intensity per field, with three fields measured per condition to ensure consistency.

### Immunofluorescence

For immunofluorescence, four animals per group were anesthetized and perfused with 0.9% saline and then 4% paraformaldehyde (PFA). The brains were postfixed in PFA (12 h, 4°C), cryoprotected overnight with 30% sucrose, and sectioned (40 μm) after freezing in OCT medium. Sections containing the ARC of the hypothalamus (bregma −1.46 mm to −2.70 mm) were used.

For immunofluorescence staining, the sections were treated with 0.3% PBS-Triton solution and blocked with 5% BSA for 1 h. The sections were then immunolabeled overnight with a combination of primary antibodies. Afterward, the sections were exposed to secondary antibodies (goat anti-rabbit IgG H&L (Alexa Fluor® 555, ab150078, Abcam) and goat anti-mouse IgG H&L (Alexa Fluor® 488, ab150078)). Counterstaining was performed with 4,6-diamidino-2-phenylindole dihydrochloride (DAPI; Sigma‒Aldrich). The slides were mounted with Prolong Diamond antifade reagent (P36970, Invitrogen). For the control procedure, the primary antibodies were omitted, but secondary antibody incubation was performed.

The primary antibodies used for staining were as follows: rabbit anti-AGRP (ab254558, 1:1,000, Abcam), rabbit anti-POMC (ab254257, 1:1,000, Abcam), rabbit anti-Iba-1 (ab178846, 1:500, Abcam), rabbit anti-CD11b (ab184308, 1:500, Abcam), mouse anti-Iba-1 (ab283319, 1:100, Abcam), mouse anti-synaptophysin1 (101011, 1:1,000, Synaptic Systems, Germany), Alexa Fluor® 488–conjugated anti-CD68 (ab201844, 1:100, Abcam), mouse anti-vGLUT2 (135421, 1:1,000, Synaptic Systems), and mouse anti-vGAT (131011, 1:1,000, Synaptic Systems).

BV2 microglia were fixed with 4% PFA for 15 min and permeabilized/blocking with 4% BSA and 0.1% Triton X-100 in PBS at 4°C for 20 min. Primary antibodies, anti-Iba-1 (10904-1-AP; Proteintech) and anti-CD68 (DF7518; Affinity Biosciences Pty Ltd., USA), were incubated overnight at 4°C. After washing, secondary antibodies (Cy3-conjugated goat anti-rabbit IgG, 1:200; 488-conjugated goat anti-rabbit IgG, 1:200, ABclonal, China) were applied for 1 h at room temperature in the dark. DAPI was used for nuclear counterstaining. Fluorescence signals were observed under a fluorescence microscope, and the intensity was quantified using ImageJ software from 7 to 10 random fields per sample.

### Golgi staining

Golgi staining was used to analyze dendritic spine morphology in the medial basal hypothalamus (MBH). Four mice per group were selected, and the brains were stained using an FD Rapid Golgi Stain™ Kit (PK401A, FD NeuroTechnologies, Inc) following the manufacturer's instructions.

### Confocal laser scanning microscopy

The optimal acquisition parameters were set at the beginning of each experiment and maintained across the immunofluorescence sections. Using a confocal microscope (TCS SP8 Xl, Leica, Germany), tissues labeled with multiple fluorescent markers were imaged. For each brain section, 2–4 microscopic fields in the MBH near the third ventricle were imaged under a 63× oil objective (N.A. = 1.4), and z-stacks of 0.5 μm sections were collected. Imaris 9.8 software (Bitplane, Switzerland) was used to process all the confocal data for 3D reconstruction. The localization of vGAT-, vGLUT-2-, and synaptophysin-immunoreactive puncta within neurons was analyzed according to the methodology of Mainardi *et al.* with modifications (32). We employed confocal laser microscopy for 3D scanning for fluorescence colocalization analysis of vGAT and vGLUT-2. 3D structures of vGAT- and vGLUT-2-immunoreactive synapses were reconstructed using Imaris 9.8 software for detailed neuronal colocalization analysis.

Synapses were reconstructed using the “Spot” function of Imaris (estimated XY diameter: 1.5 μm), adjusting the “spot quality threshold” and “minimum spot diameter” for optimal puncta detection. AgRP or POMC neurons were reconstructed using the “Cell” function of Imaris; the DAPI-positive area was considered the nucleus (nucleus diameter: 6–7 μm), and the AgRP- or POMC-positive area was considered the cytoplasm (detection type: detection of the nucleus and cell). After the cells with minimal cytoplasm and low quality were filtered out, colocalization analysis was performed on the reconstructed cells. Spots within 0 μm of the cell soma were considered in contact with the cell.

Using the “Surface” function of Imaris, microglia were reconstructed with Iba-1 as a marker. The CD68- or CD11b-positive volume within microglia was calculated, and the relative expression level of the target protein was determined as the ratio of this volume to the total volume of Iba-1–positive cells. Iba-1 average fluorescence intensity was directly calculated using the “Statistic” module of the “Surface” function. Microglial morphology, including filament length, dendritic branch number, cell body volume, and branch node number, was assessed using Imaris' “Filament” function, with convex hull and Sholl analyses performed. Separately, Golgi-stained sections were *z*-axis scanned using a confocal microscope with a 63× oil objective (N.A. = 1.4) to obtain 0.5 μm thick z-stacks. For our analysis, we specifically focused on primary dendrites located 50 μm from the soma. The investigators were blinded to the experimental conditions.

### RNA extraction and qRT–PCR analysis

Total RNA was extracted from lysates of cells subjected to different treatments using TRIzol reagent (Invitrogen, USA) following the manufacturer’s instructions. Complementary DNA (cDNA) was synthesized from the extracted RNA using a cDNA synthesis kit (Invitrogen, USA). The reverse transcription reaction was performed according to the kit protocol to ensure the accurate conversion of RNA into cDNA.

For quantitative real-time PCR, the synthesized cDNA was used as a template. The quantitative real-time PCR procedure was carried out in a thermal cycler with the following conditions: initial denaturation at 95°C for 10 min, followed by 40 cycles of denaturation at 95°C for 15 s and annealing/extension at 60°C for 45 s. Gene expression levels were quantified using the 2^−ΔΔCt^ method, with β-actin serving as the internal reference gene for normalization to minimize variations due to differing RNA input amounts or efficiencies in reverse transcription. The sequences of primers used for amplification were as follows: *CD11b*, F: 5′- GGAGCACCTCGGTATCAGCA -3′, R: 5′- CGTCCATGTCCACAGAGCAAA -3′; *C1q*, F: 5′- GGACTGGTATCCGAGGTTTTAA -3′, R: 5′- AAGCGTCATTGGGTTCTGC -3′; *C3*, F:5′- TGAACACAGCCAAAGATCGGAA -3′, R: 5′- AGCAGGCGAAACGTGGTT -3′; and *β-actin*, F: 5′- AGGGAAATCGTGCGTGAC -3′, and R: 5′- CATACCCAAGAAGGAAGGCT -3′. The data are expressed as the average of three replicates.

### Western blotting

The proteins from the ARC and cultured cells were subjected to Western blotting. The hypothalamus was isolated from four mice per group. Two of these mice were used for both Golgi staining and Western blotting, while the remaining two were used solely for Western blotting. For the mice used in both procedures, the brains were perfused with cold 0.9% saline and then bisected sagittally. One half of the hypothalamus was used for Western blotting, and the other half was used for Golgi staining. The remaining two mice were processed solely for Western blotting. The dissection boundaries for the hypothalamus were set as the optic chiasm (anterior), the lateral side of each cerebral hemisphere (lateral), and the gap between the thalamus and pons (posterior). The dissected hypothalamic tissue was approximately 1 mm thick. The tissue was rapidly frozen in liquid nitrogen and stored at −80°C. For cultured cells, proteins were extracted by discarding the medium, lysing the cells directly in 200 μl of lysis buffer, disrupting the cells with a homogenizer, and centrifuging at 12,000 r/min for 10 min. The supernatant was collected for analysis and stored at −80°C.

Proteins were extracted using homogenization buffer and centrifuged, and the supernatant was collected. After SDS‒PAGE, the proteins were transferred to 0.45 μm polyvinylidene difluoride membranes (Millipore, Burlington, Massachusetts). The membranes were incubated overnight at 4°C with the following primary antibodies: rabbit anti-PSD95 (ab238135, 1:2,000, Abcam), mouse anti-synaptophysin1 (101011, 1:1,000, Synaptic Systems), rabbit anti-C1q (ab155052, 1:500, Abcam), rabbit anti-C3 (ab97462, 1:500, Abcam), rabbit anti-CD11b (ab184308, 1:500, Abcam), anti-GAPDH (ab9485, 1: 2,500, Abcam), and anti-β-actin (bs-0061R, 1:10,000, Bioss).

The membranes were washed and incubated with secondary antibodies: HRP-conjugated goat anti-rabbit IgG H&L (bs-0295G-HRP, 1:10,000, Bioss) and goat anti-mouse IgG H&L (bs-40296G-HRP, 1:10,000, Bioss). Bands were visualized using enhanced chemiluminescence (ECL Plus; PerkinElmer, France) and imaged with a ChemiDoc MP system (Bio-Rad). Densitometric values were calculated using ImageJ (version 1.53c; National Institutes of Health), with background subtraction prior to analysis. The relative expression levels of target proteins were determined by calculating the ratio of the target protein band densitometry to the housekeeping protein band (β-actin or GAPDH).

### Statistical analysis

The data are presented as the means ± SE and were analyzed with Prism 9.0 (GraphPad Software). Differences among treatment groups were evaluated primarily via one-way ANOVA. In cases where significant heteroscedasticity was detected, Welch’s ANOVA was employed to account for unequal variances, followed by the Dunnett T3 pos thoc test for multiple comparisons. For comparisons with homogeneous variances, Tukey's pos thoc test was used after one-way ANOVA to identify significant differences among groups. Both time-based metrics, such as body weight and food intake, and blood glucose data were assessed using two-factor repeated measures ANOVA. However, while the former utilized Tukey’s pos thoc test, the latter employed the Bonferroni multiple comparison test. Owing to their hierarchical structure, data from confocal microscopy were assessed using nested one-way ANOVA. Sholl intersections of microglia were analyzed via two-way ANOVA to assess the effects of group, radius, and their interactions on the dependent variable. A statistical significance threshold was set at *P* < 0.05, and all *P* values were two-sided. The Lowess technique was employed to generate smoothed trend lines for energy intake and microglial intersections on the basis of Sholl analysis without presupposing a direct functional relationship between the examined variables.

## Results

### Body weight, energy intake, glucose metabolism and serum hormones

Throughout both the modeling and pharmacological intervention phases, no abnormal pathological signs such as vomiting, diarrhea, or dehydration were observed in the animals. Moreover, their activity levels, water intake, and general behavior remained within normal ranges.

**Fig. 1:**
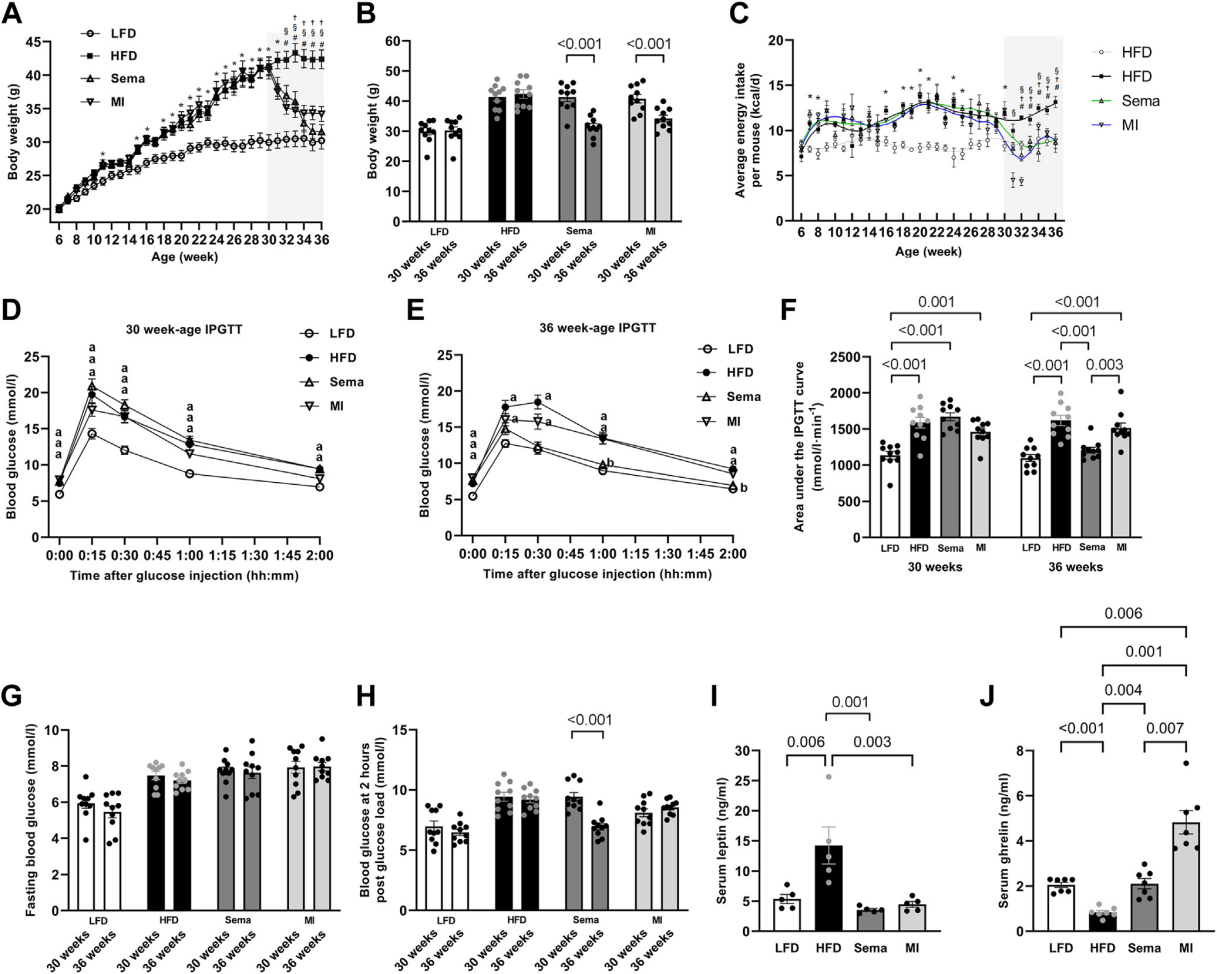
Body weight, voluntary energy intake, and metabolic profile. A: Weeks 6–30 depict the obesity induction phase, whereas weeks 31–36 (shaded area) correspond to the pharmacological intervention phase. The data were analyzed via two-factor repeated measures (RMs) ANOVA followed by Tukey’s post hoc test (n = 10). B: Analysis of body weight changes before and after pharmacological treatment. The data were processed via two-factor RM ANOVA with a Bonferroni multiple comparison test (n = 10). C: The average energy intake of the LFD, HFD, Sema, and MI groups throughout the study was analyzed. A Lowess curve facilitated the visualization of changing trends. This was processed via two-factor RM ANOVA followed by Tukey’s post hoc test (n = 5). D and E: Trajectory of blood glucose levels over time at ages 30 and 36 weeks, respectively, in accordance with the IPGTT. Significant intragroup differences are denoted by uppercase letters: “a” and “b” indicate marked differences between the LFD and HFD groups. The data were analyzed via two-factor RM ANOVA complemented by Tukey’s post hoc test (n = 10). F: Details of the area under the IPGTT curve for all groups before and after treatment. Two-factor RM ANOVA paired with Bonferroni's multiple comparison test was used for the analysis (n = 10). G and H: Contrast of the mean fasting and postmeal blood glucose levels for each group, both before and after the intervention. Analysis was carried out via two-factor RM ANOVA in conjunction with a Bonferroni multiple comparison test (n = 10). I: Serum leptin levels. Differences among groups were evaluated with ANOVA followed by Tukey's post hoc test for multiple comparisons (n = 5). J: Serum ghrelin levels. Group variations were evaluated through Welch's ANOVA due to the presence of heteroscedasticity, followed by the Dunnett T3 test for multiple comparisons (n = 7). The displayed values represent the means ± SE notations: ∗HFD = Sema = MI ≠ LFD; #HFD ≠ LFD; ✝HFD ≠ Sema; §HFD ≠ MI. HFD, high-fat diet; IPGTT, intraperitoneal glucose tolerance test; LFD, low-fat diet; MI, minocycline; Sema, semaglutide.

At 6 weeks of age, the average body weight did not significantly differ among the LFD, HFD, Sema, and MI groups (n = 10) (*P* > 0.05). At 30 weeks, all the groups presented a significant increase in body weight from baseline (*P* < 0.05). While the body weights of the HFD, Sema, and MI groups were significantly greater than those of the LFD group (*P* < 0.05), there were no significant differences among these groups (*P* > 0.05) (Fig. 1A).

After 6 weeks of pharmacological intervention, there were no significant differences in body weight among the LFD, Sema, and MI groups (*P* > 0.05), but these groups were significantly lighter than the HFD group was (*P* < 0.05) (Fig. 1A, B).

At 6 weeks, the average daily energy intake per mouse was not significantly different among the LFD, HFD, Sema, and MI groups (n = 5) (*P* > 0.05). At 7 weeks, the HFD, Sema, and MI groups had significantly greater energy intakes than the LFD group did (*P* < 0.05), but there were no significant differences among the HFD, Sema, and MI groups (*P* > 0.05). Before the 30-week pharmacological intervention, there were no significant differences in energy intake among the HFD, Sema, and MI groups (*P* > 0.05); these groups had significantly greater energy intakes than did the LFD group (*P* < 0.05). After the 6-week intervention at 36 weeks, there were no significant differences in energy intake among the LFD, Sema, and MI groups (*P* > 0.05), and the HFD group had a significantly greater energy intake than the other groups did (*P* < 0.05) (Fig. 1C).

In mice fed a HFD for 30 weeks, compared to the LFD group, glucose metabolism was impaired, as indicated by elevated fasting glucose levels (*P* < 0.05) and an increased area under the IPGTT curve (*P* < 0.05). Postintervention, the Sema group presented decreased postprandial glucose levels (*P* < 0.05) and a reduced area under the IPGTT curve (*P* < 0.05), with no significant change in fasting glucose levels (*P* > 0.05). The MI group showed no significant changes in fasting glucose levels, postprandial glucose levels, or area under the IPGTT curve (*P* > 0.05) (Fig. 1D–H).

At 36 weeks, the serum leptin levels in the HFD group were significantly greater than those in the LFD, Sema, and MI groups (all *P* < 0.05). The serum ghrelin levels in the HFD group were significantly lower than those in the LFD, Sema, and MI groups (all *P* < 0.05). The serum leptin and ghrelin levels in the Sema group were similar to those in the LFD group (all *P* > 0.05), whereas the ghrelin levels in the MI group were significantly higher than those in the LFD, HFD, and Sema groups (all *P* < 0.05) (Fig. 1I, J).

### Dendritic spine density and synaptic marker expression in the hypothalamus

**Fig. 2:**
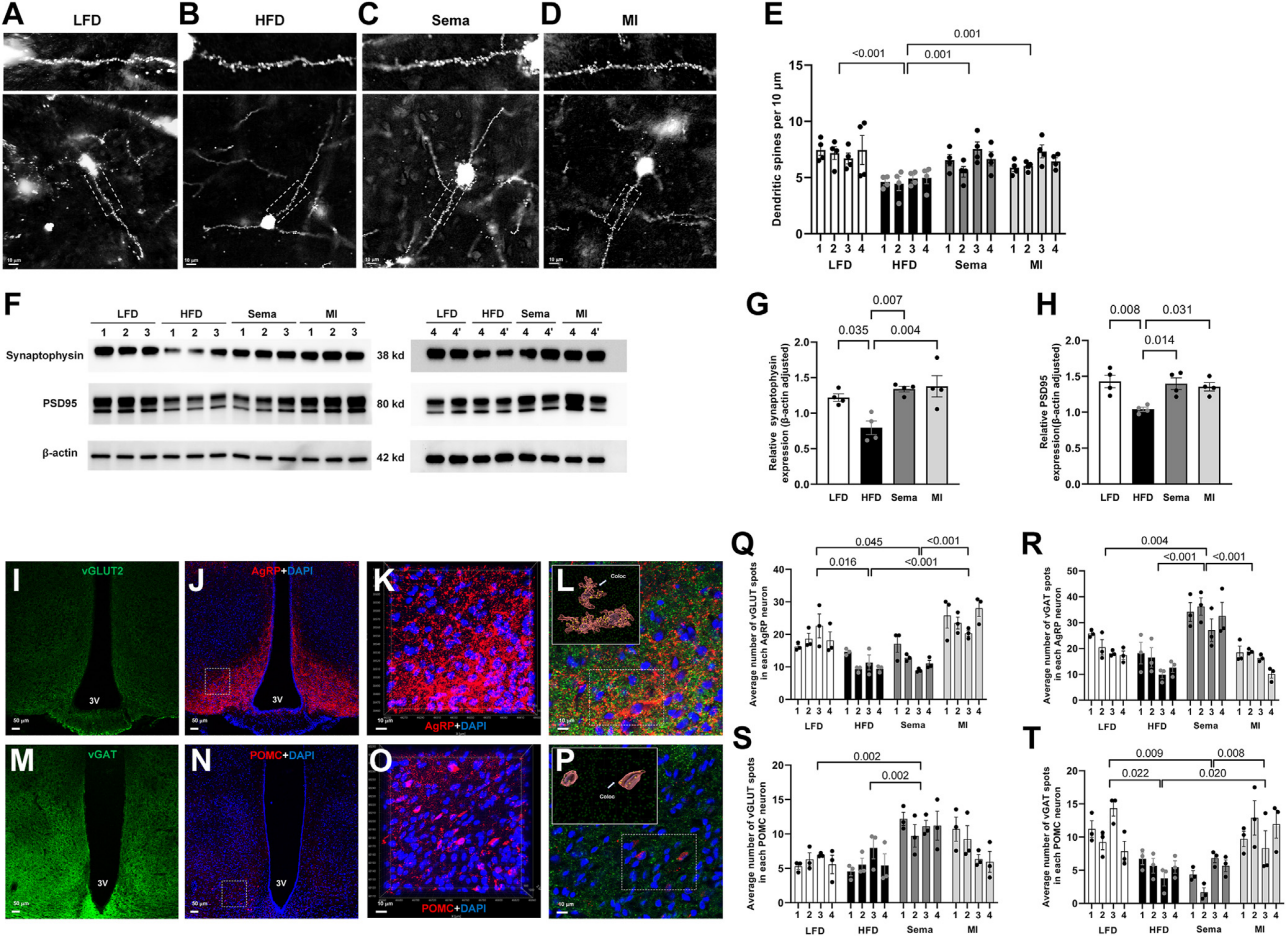
Analysis of dendritic spine density, synaptic marker expression, and synaptic input organization in the ARC of the hypothalamus. A–D: Images displaying dendritic spines in the MBH from the LFD, HFD, Sema, and MI groups, including magnified views of segments. Scale bars represent 10 μm. For clarity during spine counting, images underwent reverse coloring. E: Measurements were sourced from four animals per group, and 2–3 neurons from each of the 3–4 Golgi-stained sections were analyzed. The analyzed dendrites spanned approximately 50 μm. Nested one-way ANOVA followed by Tukey's post hoc test revealed significant differences in spine density among the groups (n = 4). F–H: Western blot analysis showing the expression levels of synaptophysin and PSD95 in the hypothalamus in each group (n = 4). Samples 4 and 4′ represent technical replicates from the same animal, and data were averaged for calculation. The data were analyzed using one-way ANOVA followed by Tukey's post hoc test. I–T: Synaptic input organization of the ARC of the hypothalamus. I: Fluorescence staining of vGLUT2 in the ARC of the hypothalamus under low magnification (10×). J: Fluorescence staining of AgRP and DAPI in the same field of view of the ARC of the hypothalamus under low magnification (10×). K: Confocal three-dimensional imaging of the area in the dotted box in (J) under high magnification (60×). L: Triple fluorescence staining image of AgRP (red), vGLUT2 (green), and DAPI (blue) in the same high-magnification field. The dotted box and the picture in the upper left corner show the 3D reconstruction of AgRP neurons and vGLUT2, and colocalization analysis was performed using object-based analysis. The yellow spots represent the colocalization of vGLUT2 with AgRP. M: Fluorescence staining of vGAT in the ARC of the hypothalamus under low magnification (10×). N: Fluorescence staining of POMC and DAPI in the same field of view of the ARC of the hypothalamus under low magnification (10×). O: Confocal three-dimensional imaging of the area in the dotted box in (N) under high magnification (60×). P: Triple fluorescence staining image of POMC (red), vGAT (green), and DAPI (blue) in the same high-magnification field of view as in (O). The dotted box and the picture in the upper left corner show the 3D reconstruction of POMC neurons and the vGAT, and colocalization analysis was performed using object-based analysis. The yellow spots represent the colocalization of vGAT with POMC. Q–T: Statistical analysis of the colocalization of excitatory synapses (vGLUT2) and inhibitory synapses (vGAT) with AgRP or POMC neurons in the hypothalamic ARC. The sections were subjected to triple fluorescence staining. Three visual fields around the third ventricle were observed via a confocal microscope. Approximately 5–10 neurons were analyzed in each visual field, representing data from four animals per group. The data were analyzed via nested one-way ANOVA followed by Tukey's post hoc test (n = 4). 3V, the third ventricle; AgRP, agouti-related peptide; ARC, arcuate nucleus; DAPI, 4,6-diamidino-2-phenylindole dihydrochloride; HFD, high-fat diet; IPGTT, intraperitoneal glucose tolerance test; LFD, low-fat diet; MBH, medial basal hypothalamus; MI, minocycline; POMC, proopiomelanocortin; Sema, semaglutide.

Significant differences in MBH dendritic spine density among the treatment groups were observed when neurons from 3 to 4 Golgi-stained sections per animal were analyzed (n = 4). Compared with the LFD, Sema, and MI groups, the HFD group exhibited a significantly lower dendritic spine density (all *P* < 0.05), with no significant differences among the latter three groups (all *P* > 0.05). Additionally, synaptophysin and PSD95 expression levels were significantly lower in the HFD group compared to the other groups (all *P* < 0.05) (Fig. 2A–H).

### Distribution of excitatory and inhibitory synapses in the hypothalamic melanocortin system

We analyzed three immunofluorescence-stained sections per animal (n = 4) and detected significant differences in the synaptic distribution in the MBH among the groups. Excitatory and inhibitory synapses were quantified using colocalization of AgRP or POMC neurons with vGLUT2 (excitatory synaptic vesicle marker) or vGAT (inhibitory synaptic vesicle marker), respectively. Compared with LFD-fed mice, HFD-fed mice showed decreased excitatory input to AgRP neurons (*P* < 0.05) and inhibitory input to POMC neurons (*P* < 0.05). Sema increased the number of inhibitory synapses on AgRP neurons (*P* < 0.05) and excitatory synapses on POMC neurons (*P* < 0.05) compared with HFD, with no change in the number of excitatory synapses on AgRP neurons or inhibitory synapses on POMC neurons (*P* > 0.05). MI increased the number of excitatory synapses on AgRP neurons (*P* < 0.05) and inhibitory synapses on POMC neurons (*P* < 0.05), with no change in the number of excitatory synapses on POMC neurons or inhibitory synapses on AgRP neurons (*P* > 0.05) (Fig. 2I–T).

### Microglial activation and phagocytic activity

**Fig. 3:**
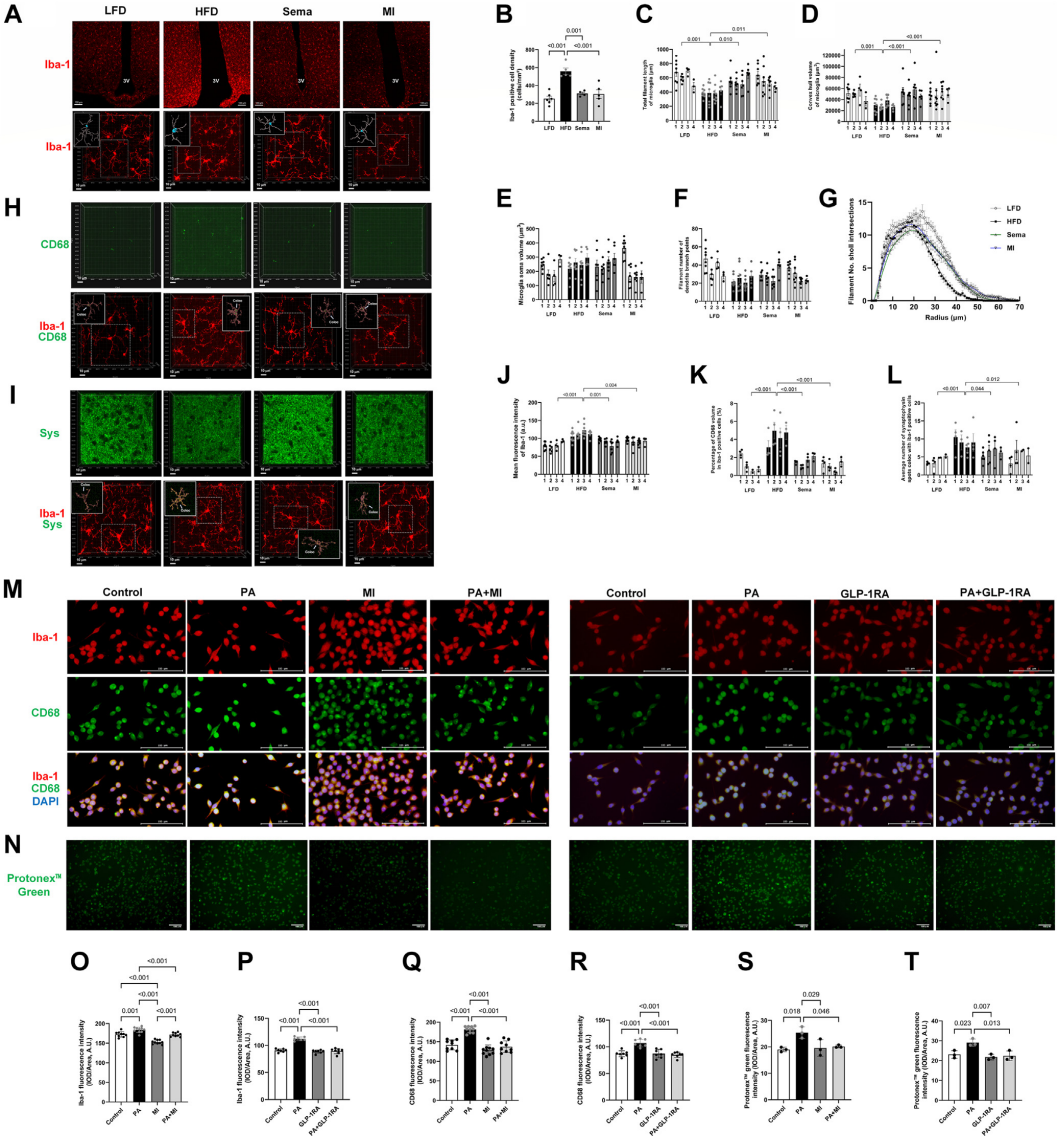
Microglial activation, morphological changes, and phagocytic activity assessment. A: Representative confocal laser scanning microscopy images of Iba-1 staining in the LFD, HFD, Sema, and MI groups are shown under low magnification (10×) and high magnification (60×). Scale bars represent 100 μm for low magnification and 10 μm for high magnification. In the upper left corner of each high-magnification panel (60×), a reconstructed filament of microglia illustrates the branches and orientations of microglial processes. 3V: the third ventricle. B: Iba-1–positive cell density in the LFD, HFD, Sema, and MI groups. The density of Iba-1-positive cells was quantified in randomly selected hypothalamic regions, including the third ventricle, under low magnification (10×). Data were collected from n = 4–6 fields per group. C–G: Analysis of the total microglial filament length, representing the intricate architecture of microglial processes; convex hull volume, representing the spatial extent of the microglial field; microglial soma volume, reflecting the size of the cell body; filament number at dendrite branch points, indicating the branching complexity; and the number of intersections based on Sholl analysis, reflecting the overall structural complexity of microglia. A total of 2–3 fields of view per slice were analyzed, with 2 slices prepared from each animal. Each group consisted of four animals. C: Total microglial filament length. Data were analyzed using nested one-way ANOVA followed by Tukey's post hoc test (n = 4). D: Convex hull volume of microglia. Data were analyzed using nested one-way ANOVA followed by Tukey's post hoc test (n = 4). E: Microglial soma volume. Data were analyzed using nested one-way ANOVA followed by Tukey's post hoc test (n = 4). F: Filament number at dendrite branch points. Data were analyzed using nested one-way ANOVA followed by Tukey's post hoc test (n = 4). G: Sholl analysis of filament number and intersections was used to assess microglial complexity relative to distance from the cell body. Two-way ANOVA revealed significant effects of treatment, radius, and their interaction. The analysis included 20 microglia per group (n = 20, total data points = 5,600). All the groups (LFD, HFD, Sema, and MI) presented an initial increase in the number of intersections, peaking at a certain distance. Beyond this peak, the HFD group presented the sharpest decline, indicating reduced branching complexity. The LFD group presented the slowest decline, maintaining more extensive branching, whereas the Sema and MI groups presented intermediate declines, suggesting partial restoration of branching in HFD-treated cells. H, I: Colocalization analysis of microglia with CD68 or synaptophysin in the LFD, HFD, Sema, and MI groups. The solid boxes represent 3D reconstructed microglial colocalization with CD68 or synaptophysin (Sys) and Iba-1, with green indicating colocalization with CD68 and yellow indicating colocalization with Sys. J: Mean fluorescence intensity of Iba-1 in microglia, with red indicating Iba-1 positivity. Data were analyzed using nested one-way ANOVA followed by Tukey's post hoc test (n = 4). K: Percentage of the CD68-positive volume occupied by microglia. The data were analyzed using nested one-way ANOVA followed by Tukey's post hoc test (n = 4). L: Average number of synaptophysin puncta per microglial cell. For each animal, 2–3 fields of view per slice were analyzed, with 2 slices per animal. Approximately 1–2 microglia in each visual field were evaluated from four animals per group. The data were analyzed using nested one-way ANOVA followed by Tukey's post hoc test (n = 4). M: Representative fluorescence images showing CD68 immunofluorescence (green) in BV2 cells subjected to different treatments. The scale bar represents 100 μm. N: Representative fluorescence images showing Protonex™ Green fluorescence (green) in BV2 cells to assess phagocytic activity under different treatments. The scale bar represents 100 μm. O, P: Iba-1 fluorescence intensity across experimental groups. Mean density was calculated as the integrated optical density (IOD) divided by the area. Statistical significance was determined using one-way ANOVA followed by Tukey's post hoc test. Data were collected from 7 to 10 randomly selected fields per group. Q, R: Quantification of mean CD68 fluorescence intensity across experimental groups. Mean density was calculated as integrated optical density (IOD) divided by area. Statistical significance was determined by one-way ANOVA with Tukey's post hoc test. Data were collected from 7 to 10 random fields per group. S, T: Quantification of mean Protonex™ Green fluorescence intensity across experimental groups. Mean density was calculated as integrated optical density (IOD) divided by area. Statistical significance was determined by one-way ANOVA with Tukey's post hoc test. Data were collected from three random fields per group. HFD, high-fat diet; IPGTT, intraperitoneal glucose tolerance test; LFD, low-fat diet; MI, minocycline; Sema, semaglutide.

HFD feeding altered hypothalamic microglial number and morphology, increasing Iba-1–positive cell density and reducing total branch length (*P* < 0.05) and convex hull volume (*P* < 0.05), while showing no effect on cell body volume or branch node number (*P* > 0.05). Sholl analysis revealed that the number of microglial intersections decreased with increasing distance from the cell body, driven by significant group, radius, and interaction effects (all *P* < 0.05). Notably, Sema and MI treatments mitigated these HFD-induced abnormalities (Fig. 3A–G).

The HFD group exhibited a significant increase in Iba-1 mean fluorescence intensity compared to the LFD, Sema, and MI groups, reflecting heightened microglial activation. There were significant group differences in the percentage of CD68-positive Iba-1–positive cells (*P* < 0.05). Compared with those in the LFD, Sema, and MI groups, the CD68-positive volume in the HFD group was increased (all *P* < 0.05), indicating increased microglial activation and phagocytic ability. Similarly, the number of synaptic puncta, represented by synaptophysin, within the microglial cytoplasm in the HFD group was greater (all *P* < 0.05), signifying increased phagocytosis of synapses by microglia. Both Sema and MI effectively counteracted these changes, reversing the increase in microglial phagocytic ability indicated by CD68 and curtailing the excessive phagocytosis of synapses by microglia (Fig. 3H–L).

Compared with the control group, the mean Iba-1 and CD68 fluorescence density in BV2 microglia stimulated with PA was significantly greater (*P* < 0.05). Similarly, the Protonex™ Green 500 Dextran fluorescence intensity, indicating phagocytic activity, showed a similar increase in response to PA stimulation. However, treatment with MI or GLP-1RA, either alone or in combination with PA stimulation, significantly reduced both the Iba-1 and CD68 fluorescence density, as well as the Protonex™ Green fluorescence intensity (*P* < 0.05), indicating a decrease in microglial activation and phagocytic activity (Fig. 3M–T).

### High-fat-induced activation of microglial phagocytic pathways and the mitigating effects of GLP-1RA and MI

**Fig. 4:**
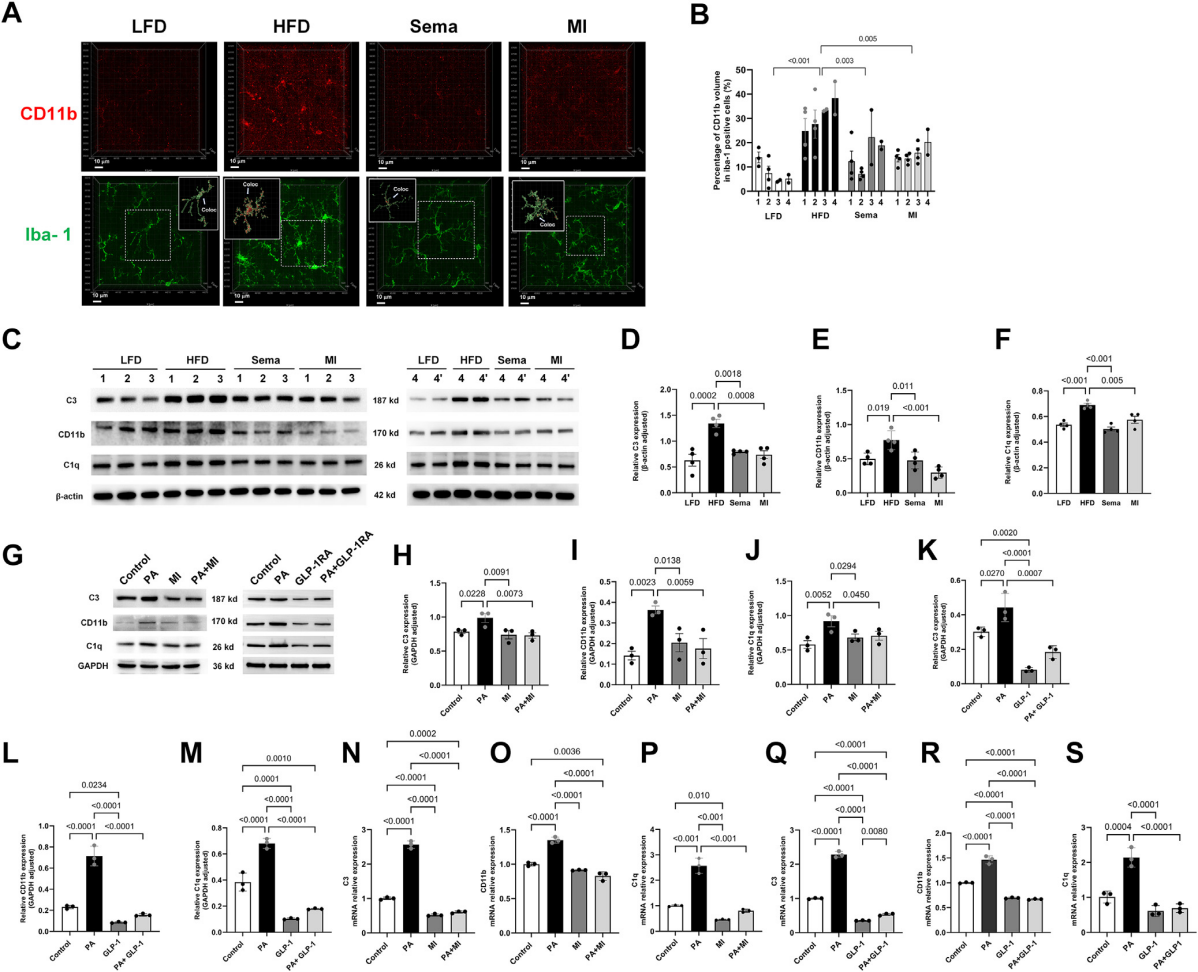
Analysis of the colocalization of microglia with CD11b and the relative expression of C3, CD11b, and C1q. A: CD11b and Iba-1 double immunofluorescence staining in the LFD, HFD, Sema, and MI groups. The solid boxes show 3D reconstructed microglial and object-based colocalization analysis of CD11b and Iba-1, with red indicating colocalization. A total of 2–3 fields of view per slice were observed, and 1 slice was prepared for each animal. Approximately 1–2 microglia in each visual field were analyzed for four animals from each group. B: Statistical analysis of the percentage of CD11b volumes among Iba-1–positive cells. The data were analyzed using nested one-way ANOVA followed by Tukey's post hoc test (n = 4). C–F: Western blot analysis of C3, CD11b, and C1q expression in the hypothalamus across different experimental groups. The data were analyzed using one-way ANOVA followed by Tukey's post hoc test (n = 4). G–S: Effects of MI or GLP-1RA on PA-induced microglial activation in BV2 cells. (G–M) Western blot analysis of C3, CD11b, and C1q expression in BV2 cells across experimental groups (n = 3 independent experiments). Statistical significance was determined using one-way ANOVA followed by Tukey's post hoc test. (N–S) qRT‒PCR analysis of *C3*, *CD11b*, and *C1q* expression in BV2 cells across experimental groups (n = 3 independent experiments). Statistical significance was determined using one-way ANOVA followed by Tukey's post hoc test. GLP-1RA, GLP-1 receptor agonist; HFD, high-fat diet; IPGTT, intraperitoneal glucose tolerance test; LFD, low-fat diet; MI, minocycline; PA, palmitic acid; qRT-PCR, quantitative real-time PCR; Sema, semaglutide.

Figure 4 illustrates the expression of proteins involved in microglial synaptic pruning. Compared with the LFD, Sema, and MI groups, the HFD group showed elevated C1q and C3 expression in the hypothalamus, suggesting that these ligands promote phagocytosis (all *P* < 0.05). In addition, increased CD11b expression and the volumetric proportion of intracellular CD11b in microglia were observed in the HFD group compared to the LFD, Sema, and MI groups (all *P* < 0.05), indicating the presence of an enhanced receptor for synaptic phagocytosis. Both Sema and MI effectively counteracted these changes (Fig. 4A–F).

Western blotting analysis demonstrated that the protein levels of C1q, C3, and CD11b were significantly elevated in the PA-treated BV2 cells compared to the control group (*P* < 0.05). Both GLP-1RA and MI treatments effectively reduced the protein levels of these markers (*P* < 0.05). In addition, PA-treated BV2 cells showed significantly increased mRNA expression levels of *C1q*, *C3*, and *CD11b* compared to the control group (*P* < 0.05). Treatment with either GLP-1RA or MI significantly decreased these mRNA levels (*P* < 0.05), indicating an inhibitory effect on the PA-induced transcriptional upregulation of phagocytosis-related genes (Fig. 4G–S).

## Discussion

Our study demonstrated that prolonged HFD exposure in mice resulted in significant metabolic dysfunctions, including obesity, impaired glucose metabolism, elevated leptin levels, and decreased ghrelin levels. Despite the increased leptin—which physiologically suppresses appetite—mice continue to consume excess energy and gain weight, indicating leptin resistance and disrupted energy homeostasis (33).

HFD-fed mice exhibited reduced dendritic spine density, decreased synaptic marker expression, and disrupted excitatory/inhibitory synaptic organization within the melanocortin system. Excitatory inputs were assessed using vGLUT2, a presynaptic vesicle marker for excitatory synapses, while inhibitory inputs were assessed using vGAT, a presynaptic vesicle marker for inhibitory synapses. Synaptic remodeling in this system is influenced by factors such as leptin, which plays a key role in hypothalamic plasticity (6, 34). Notably, HFD reduced excitatory inputs to AgRP neurons and inhibitory inputs to POMC neurons, likely reflecting compensatory mechanisms to suppress appetite (consistent with increased leptin levels) (13). However, these changes (hypothalamic structural adjustments associated with elevated leptin) did not reduce food intake or body weight, indicating impaired hypothalamic neural regulation. This reflects a form of central leptin resistance.

Our findings show that a HFD induces microglial activation in the hypothalamus, as evidenced by increased Iba-1–positive cells, elevated Iba-1 expression, and morphological changes. In HFD-fed mice, microglia exhibited increased phagocytic activity, with elevated CD68 and activation of phagocytosis-related pathways, including upregulated C1q, C3, and CD11b. We also observed increased microglial synaptic engulfment, as indicated by enhanced colocalization of microglial and synaptic markers. In vitro, PA-treated microglia showed similar increases in CD68 expression and phagocytic activity. These findings suggest that HFD-induced microglial activation and phagocytosis contribute to synaptic loss in the hypothalamus, likely impairing neuronal communication and energy homeostasis regulation.

Sema treatment markedly reduced microglial activation and synaptic phagocytosis in HFD-fed mice. The treatment normalized microglial morphology and significantly decreased the expression of phagocytic markers, including CD68, CD11b, C1q, and C3, indicating the suppression of microglia-mediated synaptic engulfment. This effect may be mediated by GLP-1 receptors expressed on microglia, which are known to exert anti-inflammatory effects upon activation (35). By attenuating excessive microglial activation, Sema protects synaptic structures and maintains neuronal integrity.

Moreover, Sema treatment restored hypothalamic synaptic marker expression and synaptic density in the ARC, as well as the synaptic balance within the melanocortin system by increasing inhibitory inputs to AgRP neurons and excitatory inputs to POMC neurons. These adjustments are essential for regulating energy homeostasis. Such changes reduce orexigenic activity in AgRP neurons and enhance anorexigenic effects in POMC neurons, shifting melanocortin system activity toward appetite suppression and improved energy expenditure. This synaptic reorganization provides a structural basis for the observed metabolic improvements.

In addition to these central effects, Sema led to significant metabolic improvements, including reduced body weight, decreased leptin levels, normalized ghrelin levels, and enhanced glucose metabolism. These findings align with previous studies demonstrating that GLP-1RA can modulate energy intake and glucose homeostasis (27, 29). By reducing microglial activation, Sema may support the hypothalamic synaptic plasticity essential for maintaining energy homeostasis under HFD conditions.

Similarly, MI treatment inhibited microglial activation and reduced synaptic phagocytosis in HFD-fed mice. This was evidenced by decreased phagocytic marker expression and the restoration of microglial morphology to a ramified, less activated state. Consistently, in vitro experiments showed that MI suppressed PA-induced microglial activation and phagocytic activity. These findings suggest that MI attenuates microglial-mediated synaptic pruning, preserving hypothalamic synaptic structures. This effect was further supported by the restoration of synaptic marker expression and density in the ARC, indicating MI's broader impact on hypothalamic synaptic integrity. Additionally, MI specifically altered the synaptic balance within the melanocortin system, increasing excitatory synapses on AgRP neurons and inhibitory synapses on POMC neurons. These synaptic adjustments create a pattern typically associated with increased orexigenic signaling and appetite stimulation.

Despite these synaptic changes, the MI-treated mice consumed less food and exhibited weight loss. This seemingly paradoxical outcome may stem from peripheral side effects of MI, such as gastrointestinal disturbances, which impair effective food intake despite heightened orexigenic signaling from the central nervous system (30). This suggests that, while the central nervous system promotes appetite through elevated orexigenic signaling and increased ghrelin levels, peripheral discomfort induced by MI may counteract these central signals, resulting in a state of passive caloric restriction. Notably, MI-treated mice presented elevated levels of ghrelin, a hormone that typically stimulates appetite, which is consistent with the observed synaptic reorganization. However, unlike Sema, MI did not improve glucose metabolism or insulin sensitivity, suggesting that its benefits are predominantly limited to weight reduction and do not extend to systemic glucose homeostasis.

Sema and MI both effectively inhibited microglial activation and reduced phagocytic activity in HFD-fed mice; however, their effects on synaptic organization and metabolic outcomes differed. Sema exhibited broader metabolic benefits, including reductions in leptin levels, normalization of ghrelin levels, and significant improvements in glucose metabolism. In contrast, MI primarily reduced body weight without enhancing glucose homeostasis or alleviating insulin resistance. These differences likely stem from the distinct pharmacological actions of the two agents beyond their shared microglial modulation.

In addition to suppressing microglial activation, GLP-1RAs, such as Sema, exert extensive metabolic regulatory effects, which may explain their superior impact on metabolic profiles beyond weight reduction. Research indicates that metabolic hormones such as leptin and ghrelin regulate compensatory synaptic reorganization within the melanocortin system (6, 7, 34), which likely plays a role in shaping microglial activity and synaptic remodeling under HFD conditions. This dual modulation of microglial activity and metabolic hormones by Sema may underpin its capacity to restore hypothalamic function and energy homeostasis more effectively than MI does.

Despite promising results, this study has several limitations. We did not assess neuronal activity or specific signaling pathways and behavioral assessments excluded energy expenditure. BV2 cells may not fully represent primary microglia. Although MI and GLP-1RA were used as pharmacological inhibitors, the definitive role of microglia in the observed hypothalamic changes remains unclear. Future studies involving microglial deletion or genetic manipulation are needed to confirm their essential role. Additionally, research should investigate how Sema and MI modulate microglial activity, particularly through GLP-1 receptor pathways, and evaluate their therapeutic effects on obesity.

## Conclusion

In conclusion, our study demonstrated that HFD-induced microglial activation is a key driver of hypothalamic synaptic impairments, contributing significantly to obesity development. Excessive microglial activity causes synaptic loss and disrupted synaptic organization within the melanocortin system, impairing hypothalamic regulation of energy balance. Treatments with Sema and MI effectively mitigated microglial activation and partially restored synaptic integrity, with Sema also providing additional metabolic benefits. These findings underscore the therapeutic potential of targeting microglial activation to address both synaptic dysfunction and broader metabolic disturbances. Understanding microglial roles in hypothalamic function is crucial for developing integrated interventions for obesity and related metabolic disorders.

## Data availability

The data that support the findings of this study are available from the corresponding author upon reasonable request.

## Supplemental data

This article contains supplemental data.

## Conflict of interest

The authors declare that they have no conflicts of interest with the contents of this article.
